# Hemolytic Disease of the Fetus and Newborn Caused by Maternal Alloanti-Fy(a)

**DOI:** 10.7759/cureus.69395

**Published:** 2024-09-14

**Authors:** Rowena D.L. Robins, Suresh Kumar I, Hari Haran A, Sahayaraj James

**Affiliations:** 1 Transfusion Medicine, Saveetha Medical College and Hospitals, Saveetha Institute of Medical and Technical Sciences, Chennai, IND; 2 Transfusion Medicine, Saveetha Medical College and Hospitals, Saveetha Institute Of Medical and Technical Sciences, Chennai, IND

**Keywords:** antenatal antibody screening, anti-fya antibodies, double surface phototherapy, duffy blood group system, hemolytic disease of fetus & newborn (hdfn)

## Abstract

Hemolytic disease of the fetus and newborn (HDFN) is commonly attributed to maternal antibodies against fetal red blood cell antigens, with anti-D being the most frequent cause. However, other antibodies, such as anti-Fy^a^ from the Duffy blood group system, can also lead to HDFN, although they are less commonly reported. This case study describes a 29-year-old woman at 38+1 weeks of gestation, with a history of multiple pregnancies and a planned elective lower-segment cesarean section (LSCS). During pre-operative testing, her blood cross-matching results were incompatible, prompting further investigation, which revealed the presence of anti-Fy^a^ antibodies. The neonate was delivered with an APGAR (appearance, pulse, grimace, activity, and respiration) score of 8/10 and 9/10 at 1 and 5 minutes, respectively, and initially exhibited no signs of severe fetal distress. However, elevated bilirubin levels were observed shortly after birth, necessitating double surface phototherapy. This case shows the clinical significance of anti-Fy^a ^in HDFN. It highlights the critical role of comprehensive antenatal antibody screening for all pregnant women, to detect potentially significant alloantibodies early and guide appropriate management to mitigate the risks associated with HDFN.

## Introduction

Hemolytic disease of the fetus and newborn (HDFN) is characterized by the destruction of red blood cells in the fetus and neonate. This destruction commonly arises from ABO incompatibility or the presence of irregular maternal antibodies transferred through the placenta [1]. These red blood cell (RBC) antibodies can develop naturally, from a previous pregnancy, or from prior transfusions. HDFN is primarily associated with maternal antibodies directed against fetal red blood cell antigens. While anti-D antibodies are the most frequently implicated in HDFN, it is crucial to recognize that a range of other red cell alloantibodies can also induce this condition, manifesting in varying degrees of severity [2].

The Duffy blood group antigen (Fy) was first identified in 1950 by Cutbush et al. This discovery was made in a patient named Duffy, who experienced hemolytic transfusion reactions following multiple blood transfusions [3]. The Duffy blood group system comprises complex, highly immunogenic glycoprotein antigens located on the surface of RBCs, as well as on endothelial cells of blood vessels, alveolar epithelial cells, renal collecting tubules, and Purkinje cells in the brain [4,5]. These antigens function as receptors for chemokines, facilitating the attraction of immune system cells. Duffy system antibodies can cause severe hemolytic transfusion reactions and HDFN [6]. In cases of severe HDFN, an exchange transfusion may be required for effective management. Here, we report a rare case of anti-Fya causing HDFN.

## Case presentation

**Figure 1:**
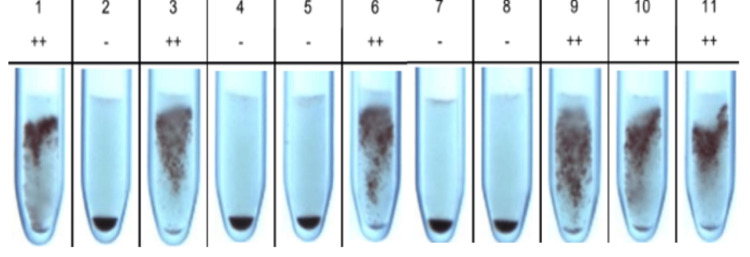
Reactivity pattern of maternal serum with the Bio-Rad ID-Dia 11-cell antibody identification panel. 2+ reactivity with the maternal serum was seen in cell lines 1, 3, 6, 9, 10, and 11.

A 29-year-old female patient with an obstetric score of G5P1L1A3, with a previous history of a lower segment cesarean section (LSCS), and currently at 38+1 weeks of gestation presented to the Obstetrics Outpatient Department for safe confinement. In view of her previous poor obstetric history, the patient was planned for an elective LSCS. On admission, a non-stress test and fetal Doppler were performed to ensure fetal well-being, and both were normal. The mother’s routine investigations were sent to the laboratory. Our blood center received a request for blood group typing and cross-matching for packed red blood cells with the appropriate blood samples. Blood grouping was performed using gel column agglutination with Bio-Rad Diaclon, and the patient's blood group was found to be A RhD-positive. The cross-matching procedure was conducted using Bio-Rad column agglutination gel cards (Anti-IgG + Anti-C3d cards), and cross-matching was found to be incompatible. A total of six units were crossmatched and typed until a compatible unit was identified. A direct Coombs test and autocontrol were also performed using Bio-Rad column agglutination gel cards and were found to be negative. An indirect Coombs test was performed, which was positive and indicated the presence of an alloantibody. A detailed immunohematological workup was conducted to identify the alloantibody present in the patient’s serum. Antibody screening and identification were performed using Bio-Rad ID Dia cell I, II, III Asia (Mia+) 3-cell panel (lot number 907084.56.2) and Bio-Rad ID Dia panel 11x4 (lot number 894233.87.1), respectively. The findings of the antibody identification panel are shown in Figure 1.

**Figure 2:**
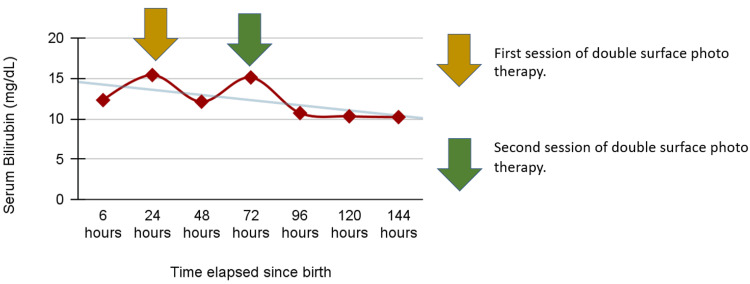
Graph depicting the increase in serum bilirubin levels and timing of double surface phototherapy intervention.

2+ cell reactivity was found in cell lines 1, 3, 6, 9, 10, and 11 of the Bio-Rad ID Dia 11-cell panel. Elucidation was done using the antigram provided by the manufacturer, and anti-Fy^a^ was identified in the maternal serum. Phenotyping of the mother’s red blood cells using anti-Fy^a^ serum revealed that she was Fy^a-b+^. Titration was conducted to assess the level of anti-Fy^a^ antibodies in the maternal serum, which was found to be 8. This was performed using a semi-quantitative, double-dilution method with pooled in-house Fy^a+^ red cell suspension. Phenotyping of the paternal RBC sample indicated the presence of Fy^a+b+^ antigens. The planned elective LSCS was carried out, and a live female baby weighing 2.75 kg was delivered. The APGAR (appearance, pulse, grimace, activity, and respiration) score was 8/10 at one minute and 9/10 at five minutes. Upon examination, the baby appeared pink, alert, and showed no signs of hydrops. Systemic examination also revealed no abnormalities. Due to the detection of irregular antibodies in the maternal serum, a cord blood sample was collected for testing. Total blood count, serum bilirubin, blood grouping and typing, and direct Coombs test were performed. The baby’s blood group was found to be A RhD positive. The DCT, performed using a polyspecific (Anti-IgG + Anti-C3d) anti-human globulin Coombs gel card, showed a result of 2+. The baby’s RBCs were phenotyped by direct agglutination with anti-Fy^a^ and anti-Fy^b^ reagents, revealing a phenotype of Fy^a+b+^. Adsorption and elution were performed on the cord blood cells. Antibody identification using the Bio-Rad ID Dia 11-cell panel (894233.87.1) was done on the prepared eluate, confirming the presence of anti-Fy^a^. Serum bilirubin levels were elevated on the first day of life, so the baby was transferred to the NICU (neonatal intensive care unit). To prevent complications, double surface phototherapy was initiated. Serial monitoring of bilirubin levels was performed. Figure 2 illustrates the increasing trend in serum bilirubin and the timing of intervention with double surface phototherapy.

At birth, the newborn’s serum bilirubin level was 12.3 mg/dL (normal range: 2-6 mg/dL). After 24 hours, it increased to 15.4 mg/dL. To address the rising bilirubin levels and prevent complications, double surface phototherapy was initiated 24 hours after birth, with regular monitoring of bilirubin levels. Following the initial phototherapy session, the bilirubin levels gradually decreased to baseline but then rose again to 15.1 mg/dL. Consequently, a second round of double surface phototherapy was administered 72 hours after birth. By 96 hours, bilirubin levels had decreased to below the baseline threshold of 11 mg/dL. Subsequent monitoring showed that bilirubin levels remained within safe limits, and there was no evidence of bilirubin-induced neurological dysfunction (BIND). The baby’s parents were advised to consistently attend scheduled follow-up appointments to ensure thorough evaluation and assessment of the baby’s growth and development.

## Discussion

ABO incompatibility has become the primary cause of HDFN in the Western world, while in many developing countries, Rh incompatibility remains a common cause. In addition to anti-D antibodies, cases of moderate to severe HDFN associated with other alloantibodies have been reported in Asian countries over the last decade [7].

The Duffy antigen system consists of two antigens, Fy^a^ and Fy^b^, which are inherited through codominant alleles, resulting in four distinct phenotypes: Fy^(a+b-)^, Fy^(a-b+)^, Fy^(a+b+)^, and Fy^(a-b-)^[8]. The Duffy gene (DARC) is located on chromosome 1, and its expression is determined by three allelic variants: Fy^a^, Fy^b^, and a silent gene, Fy [9]. 

The prevalence of Duffy blood group system antigens varies significantly across different populations. In the Indian population, the prevalence of the Fy^a ^antigen is 87.4%, while the prevalence of the Fy^b ^antigen is 57.6% [10]. Anti-Fy^a^ and anti-Fy^b^ are IgG antibodies that develop in individuals lacking the respective antigens following exposure through transfusion or pregnancy. These antibodies can be detected using the indirect antiglobulin test technique with antigen-positive cells. Anti-Fy^a^ antibodies are relatively common, comprising 6-10% of clinically significant antibodies detected by immunohematology laboratories. The presence of these antibodies can result in both immediate and delayed hemolytic transfusion reactions varying in severity from mild to severe hemolysis [11]. Anti-Fy^b^ antibodies are not linked to HDFN. However, a review of published data on HDFN secondary to anti-Fy^a^ antibodies revealed a neonatal mortality of 18%, with nearly a third of the affected neonates requiring an exchange transfusion [8,12]. Even low maternal antibody titers, such as eight, were found to be associated with moderate HDFN requiring exchange transfusion. In the cases where the paternal genotype is heterozygous, fetal PCR typing can be performed using amniocentesis [13]. 

A comprehensive review of the literature provides crucial insights into managing pregnancies affected by anti-Fy^a^ antibodies. It emphasizes the importance of assessing the risks associated with varying antibody titers. Although high titers are typically concerning, lower titers also warrant attention [14]. This case evaluates the necessity for a universal antibody screening program for red cell antibodies in the first trimester of pregnancy. Their detection is critical in predicting the severity of HDFN. Evidence from documented cases indicates that standard antenatal monitoring for expectant mothers with alloimmunization, such as the evaluation of middle cerebral artery peak systolic velocity, does not effectively determine the severity of the condition. Furthermore, studies highlight that the failure to identify irregular antibodies, like Anti-Fy^a^, can result in severe HDFN and potential stillbirth. This oversight may also contribute to significant fetal health issues that require interventions such as double volume exchange transfusion and intensive phototherapy to manage the situation effectively [14]. There is a lack of extensive long-term studies assessing severity of alloimmunization during pregnancy, the clinical significance of non-D antibodies and intervention approaches in the Indian population. Large-scale studies involving pregnant women are necessary to gather substantial evidence for formulating guidelines on testing and intervention strategies for alloimmunization during pregnancy [15].

Published data reveal that Anti-Fy^a^ antibodies produce severe HDFN, requiring intrauterine transfusions, exchange transfusions, and high-intensity phototherapy, and can cause fetal hydrops and stillbirth [16]. In our case, the baby did not develop such complications. However it is necessary for the parents to stay vigilant with regular follow-ups.

## Conclusions

This case highlights the importance of blood group antigens beyond the Rh system, demonstrating their potential role in causing HDFN. It also underscores the significance of integrating comprehensive antenatal antibody screening into routine antenatal investigations through the use of screening cell panels. This approach facilitates the detection of clinically significant minor blood group antibodies present in the maternal serum, which could lead to HDFN. Early identification of these antibodies enables obstetricians to anticipate the risk of HDFN, allowing for timely monitoring and intervention, where feasible. Mothers found to be alloimmunized should be closely monitored for maternal antibody titers and fetal well-being throughout pregnancy. Furthermore, planning for the management of HDFN in a newborn with positive maternal antibodies should be coordinated prior to delivery. This can ensure comprehensive and thorough prenatal care for all antenatal women. Implementing such screening practices can improve outcomes by preventing complications associated with undetected alloimmunization. 
